# Stability and Photothermal Properties of Fe_3_O_4_-H_2_O Magnetic Nanofluids

**DOI:** 10.3390/nano13131962

**Published:** 2023-06-28

**Authors:** Chengya Zhang, Lei Gao, Xiaofeng Zhou, Xiaohu Wu

**Affiliations:** 1 School of Physical Science and Technology & Jiangsu Key Laboratory of Thin Films, Soochow University, Suzhou 215006, China; 2 School of Optical and Electronic Information, Suzhou City University, Suzhou 215104, China; 3 College of Science, Hohai University, Nanjing 210024, China; 4 Shandong Institute of Advanced Technology, Jinan 250100, China

**Keywords:** magnetic nanofluids, droplet–droplet mixing technique, photothermal conversion efficiency

## Abstract

Solar collectors are more efficient and commercial devices for collecting solar energy, compared to other solar energy utilizations. To improve the efficiency of solar collectors, it is important to prepare a liquid heat-collecting medium, which is stable and has high photothermal properties. Therefore, in this work, we develop a droplet–droplet mixing technique to prepare Fe_3_O_4_-H_2_O magnetic nanofluid. The results show that magnetic nanofluids prepared using the droplet–droplet mixing technique have more stable performance and a better encapsulation of dispersants than those prepared via traditional liquid–liquid mixing. Then, the thermal conductivity and photothermal properties of Fe_3_O_4_-H_2_O magnetic nanofluids are investigated experimentally and theoretically. The thermal conductivity and temperature of the magnetic nanofluid with Fe_3_O_4_ nanoparticles of a 1.0% volume fraction can reach the maximum value of 0.95 W/m∙K and 73.9 °C when the magnetic field strength is equal to the saturation magnetic field of 800 Gs. These findings provide insights into the potential applications of Fe_3_O_4_-H_2_O magnetic nanofluids in direct absorption solar collectors, heat exchangers, automobile radiators, etc.

## 1. Introduction

With the rapid development of science and technology, scientists put much effort into finding solutions to renewable energy demands [1]. Solar energy is developed as a renewable energy source due to its abundant, free, and clean nature [2]. The solar collector is one of the applications of solar energy [3,4]. To increase collector efficiency, the direct absorption collector is proposed. In this regard, the liquid heat-collecting medium is a crucial factor affecting the heat collection efficiency of the direct absorption collector [5].

As one kind of liquid heat-collecting medium, nanofluid is a suspension of nanoparticles in the base fluid [6]. It has been considered a new-type heat transfer fluid because of its enhanced thermal conductivities [7,8]. When the nanoparticles are magnetic materials such as iron, cobalt, nickel, and their oxides, the nanofluid is referred to as magnetic nanofluid [9]. Under the influence of the magnetic field, the nanoparticles interact with each other via dipole–dipole interaction, resulting in chain-like clusters in the magnetic nanofluid. Consequently, the magnetic nanofluids with chain-like clusters can exhibit better thermal physical properties and photothermal performance under a magnetic field [10]. For instance, Zhu et al. [11] attributed the abnormal thermal conductivity of Fe_3_O_4_ nanofluids to nanoparticle clustering and alignment. In addition, Philip et al. [12] presented the tunable thermal conductivity of magnetic nanofluids by controlling the applied magnetic field strength. Furthermore, it was found that thermal conductivity is reversible under repeated magnetic cycling. Altan et al. [13] observed that the thermal conductivity of Fe_3_O_4_ magnetic nanofluids could be enhanced at low-magnetic-field strengths due to thermomagnetic convection. Ebrahimi et al. [14] found that the rate of thermal conductivity growth in Fe_3_O_4_/CuO nanofluid was strongly correlated with the magnetic field strength. Moreover, many scientists studied the influence of the applied magnetic field on the photothermal properties of magnetic fluids. Liu et al. [15] showed that the photothermal conversion efficiency of Fe_3_O_4_ magnetic fluid in the diluted volume fraction of 0.05% was increased by 6% to that of the base liquid. Wang et al. [16] demonstrated that the photothermal conversion efficiency of the RGO/α-Fe_2_O_3_ nanofluid was increased by 14.5% compared to that in the absence of an external rotating magnetic field. Shin et al. [17] observed that the photothermal conversion efficiency of MWCNT/Fe_3_O_4_ hybrid fluid increased with the magnetic field strength due to the improved specific surface area and the chain-like structure of Fe_3_O_4_ nanoparticles. Shojaeizadeh et al. [18] found that when the magnetic field strength was 1.2 T, the collector’s conversion efficiency of Mn-Zn Fe_2_O_4_ fluid was increased by 26.8% in contrast to that without the magnetic field. Later, water-based fluid with Fe_3_O_4_ nanoparticle-decorated carbon nanotubes was investigated for photothermal conversion applications [19]. The nanotubes became straight and aligned under the action of the weak applied magnetic field, which enhanced the photothermal conversion performance. However, with a further increase in the magnetic field strength, the nanotubes formed bulk nanotubes via large magnetic attraction, which weakened the photothermal conversion performance.

As is known, magnetic nanofluids are synthesized via various physical and chemical processes [20,21], and there are two methods including the one-step or two-step method to prepare nanofluids. The one-step method combines the manufacturing of nanoparticles and the synthesis of magnetic nanofluids in one step [22]. In this process, the magnetic nanoparticles are formed and dispersed in the liquid phase, so solid handling processes such as drying, transportation, dispersion, storage, and stabilization are avoided [23]. Additionally, the one-step method can prevent the oxidation of nanoparticles and minimum agglomeration. However, the method has the drawback of a slow production process. In addition, it is restricted only to low-pressure fluids and low concentrations of nanoparticles. For the two-step method, the first step is to prepare magnetic nanoparticles with the chemical co-precipitation method, and the second one is to disperse the nanoparticles into a base fluid via ultrasonication [24]. For instance, Bagheli et al. [25] used the chemical co-precipitation technique to synthesize Fe_3_O_4_ nanoparticles. The nanoparticles were dispersed into the base fluid using tetramethyl ammonium hydroxide and intensive ultrasonic vibration as a surfactant. For the second step of the two-step method, Wang et al. [26] dispersed the Fe_3_O_4_ nanoparticles into water with an ultrasonic vibrator. Han et al. [27] made Fe_3_O_4_ nanoparticles functionalized with carboxyl and amine groups as a polymer compound to prevent aggregation. Actually, the two-step method is of interest to many researchers due to its simplicity. However, this method has drawbacks too. At first, clustering and agglomeration easily occur as the particle possesses a high surface energy. Secondly, fast sedimentation may arise due to gravitational effects.

Among the magnetic nanofluid family, the Fe_3_O_4_ nanofluid is one of the exciting and well-known constructed fluids. Furthermore, Fe_3_O_4_ nanoparticles are readily available and cost-effective compared to other types of nanoparticles. These make them an attractive choice in the development of efficient nanofluids. As mentioned above, in the second step of the two-step method, Fe_3_O_4_ nanoparticles are directly dispersed into the base fluid via ultrasonication. Essentially, Fe_3_O_4_ nanoparticles and the base fluid are mixed in liquid–liquid form. In this paper, to increase the wrapping probability between dispersant and particles and improve the stability of the magnetic nanofluids, the solution of Fe_3_O_4_ nanoparticles and the dispersants are, respectively, changed from the form of liquid to droplet using a peristaltic pump and ultrasonic atomizer. In the end, two kinds of droplets are mixed via ultrasonication. We shall show that since our technique changes the dispersion pattern from ordinary liquid–liquid mixing to droplet–droplet mixing, the prepared magnetic nanofluids exhibit quite stable performance. After that, the thermal conductivity and the photothermal conversion efficiency of the magnetic nanofluids under the magnetic field’s action are studied both theoretically and experimentally, and there is an optimal magnetic field strength to realize the maximal thermal conductivity or the maximal photothermal conversion efficiency. Our study may offer the direction for the design of nanofluids that exhibit outstanding photothermal performance, and is helpful for engineering applications in solar thermal collectors, heat exchangers, automobile radiators, electronic devices, and so on [28,29].

## 2. Experimental Setup

### 2.1. Preparation of Fe_3_O_4_-H_2_O Magnetic Nanofluids

In this paper, we adopted the two-step method to prepare Fe_3_O_4_-H_2_O magnetic nanofluids. For the first step, Fe_3_O_4_ nanoparticles were prepared using the chemical co-precipitation method. At 85 °C, 12.4 g of FeCl_3_·6H_2_O and 5.2 g of FeCl_2_·4H_2_O were dissolved in 50 mL of water with vigorous stirring. Then, 26.5 mL of NH_3_·H_2_O and 2 mL of oleic acid were added dropwise into the solution for 1 min until the pH reached about 9.5, and the solution was continuously stirred at 85 °C for 20 min. After that, the solution was kept still at 85 °C for 30 min. Ultimately, the prepared particles were washed with deionized water and ethanol. In the above process, Fe_3_O_4_ nanoparticles were prepared using the chemical reaction. The chemical equation for this process can be expressed as follows:

(1) $$2{\mathrm{FeCl}}_{3}\cdot 6H_{2}O+{\mathrm{FeCl}}_{2}\cdot 4H_{2}O+8{\mathrm{NH}}_{3}\cdot H_{2}O={\mathrm{Fe}}_{3}O_{4}+8{\mathrm{NH}}_{4}\mathrm{Cl}+20H_{2}O.$$

**Figure 1:**
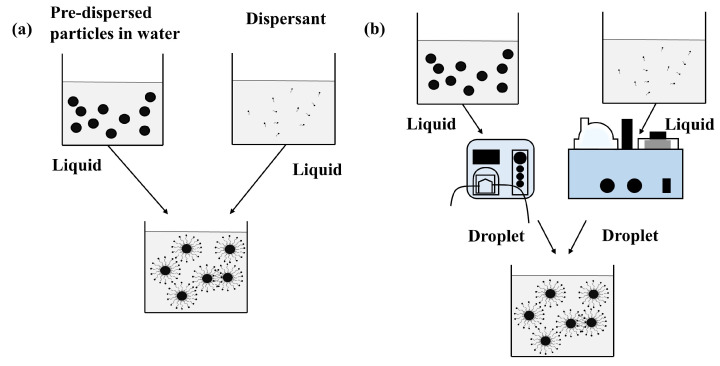
Comparison of the mixing technique in the second step, (**a**) the traditional liquid–liquid mixing technique and (**b**) the droplet–droplet mixing technique.

For the second step, magnetic nanofluids are were prepared by directly stirring the dispersant and the solution containing particles (pre-dispersed particles in the base fluid) in the liquid–liquid form [30], as shown in Figure 1a. This paper employed the droplet–droplet mixing technique to prepare magnetic nanofluids (see Figure 1b). In detail, a solution of the dispersant, sodium dodecyl benzene sulfonate (SDBS) in water, was prepared and then placed into an ultrasonic atomizer (WH–2000, Yuehua Medical Equipment Factory Co., Ltd., Shantou, Guangdong, China). After a few minutes, liquid containing SDBS became the droplets of SDBS. At the same time, magnetic particles were dissolved in water. After that, such liquid containing magnetic particles was placed in a peristaltic pump (KSP-F01A-STP, KaChuanEr Fluid Technology Co., Ltd., Shanghai, China), which pumped droplets composed of magnetic particles. After two kinds of droplets were mixed, we observed magnetic particles coated with the dispersant. In the end, magnetic nanofluids were prepared. With the droplet–droplet mixing technique, the contact area between the magnetic particles and the dispersant increased, hence the wrapping probability becoming large. As a consequence, magnetic nanofluids involved in the droplet–droplet mixing technique may exhibit good stability and superior photothermal performance in comparison to those involved in the traditional liquid–liquid mixing technique.

### 2.2. XRD and SEM Pattern of the Sample

**Figure 2:**
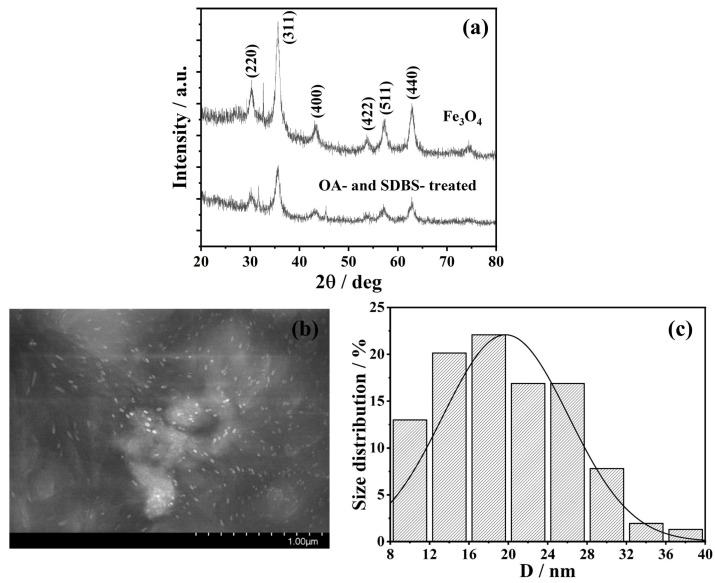
Characterization diagram of Fe_3_O_4_ nanoparticles. (**a**) XRD pattern; (**b**) SEM pattern; and (**c**) size distributions of Fe_3_O_4_ nanoparticles with different sizes.

The X-ray diffraction (XRD, D/MAX-III-B-40KV) and scanning electron microscope (SEM, SU8100, Hitachi) patterns of the synthesized nanoparticles are presented in Figure 2. Figure 2a indicates that the diffraction peaks observed at *2θ* = 30.15°, 35.5°, 43.2°, 53.5°, 57.1°, and 63.9° corresponded to the (220), (331), (400), (422), (511) and (440) crystal planes of the Fe_3_O_4_, respectively. The positions of the characteristic peak of Fe_3_O_4_ remained unchanged before and after coating, indicating that the structure of particles was maintained. In Figure 2b, the bright spots represent Fe_3_O_4_ particles. It is evident that the overall distribution of the particles was homogeneous, and there were a few large particles due to the aggregations of small particles. In addition, the particles were spherical or near-spherical in shape, and the particle size distribution is shown in Figure 2c. It can be seen that the size of Fe_3_O_4_ particles was in the range of 8–40 nm. Additionally, the probability density function was followed by the normal distribution, $f(D)=Ae^{-\frac{{\left(D-\mu \right)}^{2}}{2\sigma^{2}}}$, with the mathematic expectation μ=19.72 and the standard deviation of σ=6.39. Additionally, the average size of the particles is about 20 nm in statistical analysis.

### 2.3. Stability of Fe_3_O_4_-H_2_O Magnetic Nanofluids

**Figure 3:**
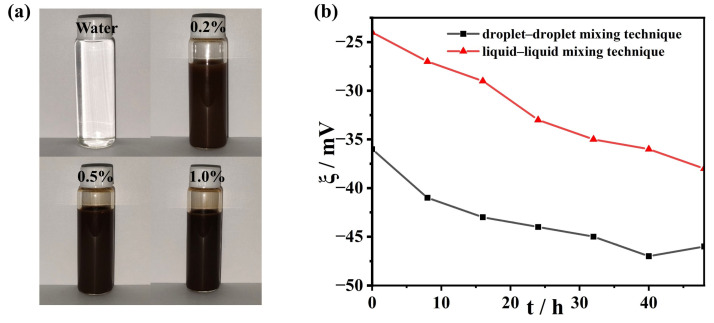
Stability of magnetic nanofluids. (**a**) The magnetic nanofluids with different volume fractions and (**b**) zeta potential versus time for magnetic nanofluids.

The visualization technique [30,31] and zeta potential analysis (ZetaPALS-1) are effective methods for analyzing the stability of magnetic nanofluids. Figure 3a shows the samples of the magnetic nanofluid with volume fractions of 0.2%, 0.5%, and 1%. No significant precipitation was observed, and the prepared magnetic nanofluids with the droplet–droplet mixing technique were very stable even for 30 days. However, the magnetic nanofluid prepared using the liquid–liquid technique (the traditional method) could only be stable for about two weeks. At the same time, the zeta potential, ξ, of the magnetic nanofluids is measured, as shown in Figure 3b. For the magnetic nanofluids including the droplet–droplet technique, the absolute zeta potential values were more significant than 45 mV after 48 h, representing good stability. A high zeta potential indicates strong repulsion between nanoparticles, thus depicting good stability [32]. A powerful surface charge is created on the nanoparticle, preventing nanoparticle aggregation. Meanwhile, the lower zeta potential is observed for a magnetic nanofluid with the liquid–liquid mixing technique [see Figure 3b]. Incidentally, the Fe_3_O_4_-H_2_O magnetic nanofluid was prepared using the liquid–liquid mixing technique, and the absolute values of the zeta potential [33] were also found to be less than those in our results. Therefore, we conclude that the droplet–droplet mixing technique yields better stability than the traditional one does when it involves the preparation of magnetic nanofluids.

### 2.4. Response of Magnetic Nanofluids in the Presence of the Magnetic Field

**Figure 4:**
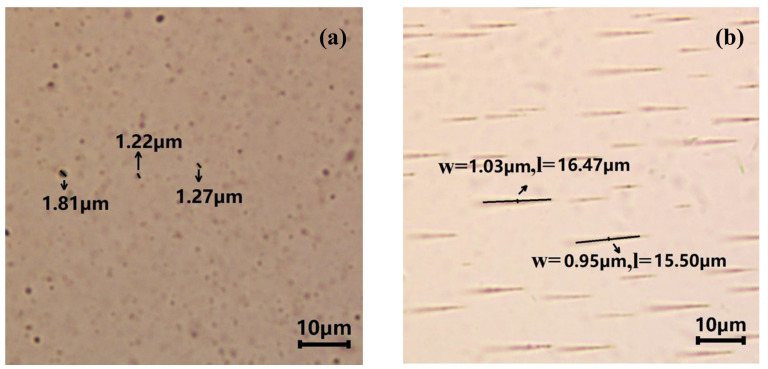
Effects of the magnetic field on the distribution of magnetic particles (**a**) without the magnetic field and (**b**) with the magnetic field; *l* is the length of the chain-like aggregate, and *w* refers to the width of the chain-like aggregate.

In this subsection, the polarization microscope (MV3000R/TR) is adopted to observe the aggregates of the nanoparticles in magnetic nanofluids. Figure 4 shows the distributed states of the magnetic particles in the absence and presence of the magnetic field. Without the magnetic field, the aggregates of Fe_3_O_4_ particles are distributed in a homogeneous manner, whose shape is almost spherical with a size of 1–2 μm (see Figure 4a). On the other hand, Figure 4b shows the response of magnetic nanofluids in the presence of the magnetic field. When the magnetic field strength of about 700 Gs is applied, the chain-like structures are formed along the direction of the applied magnetic field, and the length (width) of the aggregates, *l* (*w*), is about 10–16 μm (0.5–1.5 μm) (see Figure 4b). The chain-like aggregates can return to their initial states after removing the magnetic field. This demonstrates that the droplet–droplet mixing technique makes the nanofluids have good stability, and the addition of the magnetic field does not destroy the dispersion effect of the dispersants. It is evident that under the influence of the applied magnetic field, the chain-like structures can lead to the excellent conduction of heat, resulting in a significant enhancement of the effective conductivity and hence good photothermal properties of the magnetic nanofluids.

### 2.5. Measurement of Thermal Properties and Photothermal Properties

**Figure 5:**
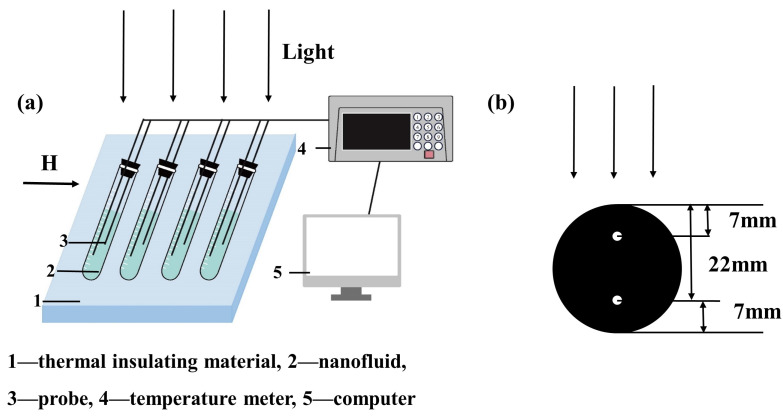
(**a**) The schematic diagram for measuring the photothermal property and (**b**) the cross-section of the test tube. One probe is located at 7 mm, and another probe is located at 22 mm.

To explore the thermal characteristics of Fe_3_O_4_-H_2_O magnetic nanofluids, thermal conductivity was measured using a thermal conductivity meter (HCDR-S, Huicheng Instrument Co., Ltd., Nanjing, China) based on the principle of the transient hot wire method [34]. The precision of the thermal conductivity meter was ±3%, the measuring range of the thermal conductivity meter was 0.005–300 W/m·K, and the repeatability error of the instrument was ≤3%. On the other hand, Figure 5a illustrates the schematic diagram of the system for the measurement of the photothermal property. The test tubes made of transparent glass were used to hold Fe_3_O_4_-H_2_O magnetic nanofluids; the diameter of the test tube was 3 cm, and the height was 15 cm. The liquid level of the four test tubes was kept at the same height to ensure identical aperture areas. The experiment was conducted under an artificial light source with an irradiation intensity of 900 W/m^2^. A set of magnetic steel was placed near the test tube to adjust the strength of the magnetic field between 0 and 1000 Gs for testing the response of the magnetic nanofluids. The temperature of the magnetic nanofluids was measured using a temperature meter (DC5508U, Zhongshan Zhongxiang Instrument Co., Ltd., Guangdong, China). The temperature measurement range of the temperature meter was −100–260 °C, and the precision of the instrument was ±0.2%. Moreover, the temperature of the magnetic nanofluids could be obtained every five seconds. Figure 5b shows the cross-section of the test tube. Probes were placed at different distances in the tube to measure the temperature of the magnetic nanofluids. One probe was located 7 mm from the light plane (near the light end, *h* = 7 mm), and another probe was located 22 mm from the light plane (far from the light end, *h* = 22 mm).

## 3. Results and Discussion

### 3.1. Thermal Properties

The magnetic nanofluid exhibits an obvious change in thermal conductivity under the influence of the magnetic field. In the absence of an applied field for theoretical investigation, Bruggeman’s effective medium theory is used to predict thermal conductivity [35]. When the magnetic field is applied, the magnetic nanoparticles are aligned with chain-like clusters parallel to the direction of the field (see Figure 4), and these clusters form a more efficient pathway for heat conduction [36]. In this regard, we used the homogenization method to predict the effective thermal conductivity of the Fe_3_O_4_-H_2_O magnetic nanofluids, which can be described as follows:

(2) $$f_{cluster}\frac{k_{cluster}-k_{nf}}{k_{nf}+A_{c}(k_{cluster}-k_{nf})}+(1-f_{cluster})\frac{k_{m}-k_{nf}}{k_{nf}+A_{c}(k_{m}-k_{ex})}=0,$$

(3) $$f_{cluster}\frac{k_{cluster}-k_{ex}}{k_{ex}+A_{a}(k_{cluster}-k_{ex})}+(1-f_{cluster})\frac{k_{m}-k_{ex}}{k_{nf}+A_{a}(k_{m}-k_{ex})}=0,$$

where *f_cluster_* and *k_cluster_* represent, respectively, the aggregated clusters’ volume fraction and thermal conductivity. *A_a_* (*A_c_*) is the depolarization factor of the chain-like aggregated clusters in the magnetic field concerning orientations perpendicular to (or parallel to) the applied magnetic field. *k_m_* is the thermal conductivity of the host medium. In addition, *k_ex_* is the effective thermal conductivity perpendicular to the applied field. At the same time, *k_nf_* is the one parallel to the applied magnetic field measured in our experimental system. Here, we describe the thermal conductivity of the clusters, *k_cluster_*, under the magnetic field using the differential effective medium theory [37].

(4) $$\frac{k_{cluster}-k_{p}}{k_{m}-k_{p}}{\left(\frac{k_{m}}{k_{cluster}}\right)}^{1/3}=1-f_{\mathrm{int}},$$

where *k_p_* is the thermal conductivity of magnetic nanoparticles. *f_int_* represents the volume fraction of magnetic particles within the chain-like aggregated cluster. Hence, one yields the volume fraction of the magnetic particles in the nanofluids, *f = f_cluster_f_int_*.

When the applied magnetic field is almost in the saturation state, numerous magnetic particles gather together in a chain-like manner. Consequently, *f_int_* is expected to be quite large and is chosen to be 0.92 here. Using Equation (4), we obtain *k_cluster_* = 52.04 W/m·K. Note that without the applied magnetic field, we have *f = f_cluster_* and *f_int_* = 1. Then, the thermal conductivity, *k_nf_*, of magnetic nanofluids can be calculated theoretically under the external magnetic field. One refers the readers to Ref. [36] for a more detailed discussion. On the other hand, the thermal conductivity, *k_nf_*, of magnetic nanofluids with volume fractions of 0.2%, 0.5%, and 1.0% is also measured using a transient hot wire method.

**Figure 6:**
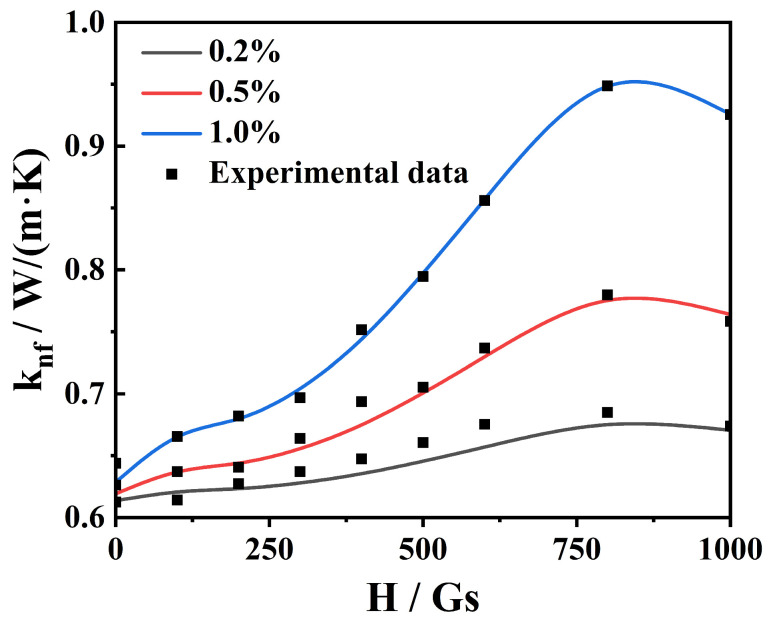
Influence of the magnetic field on the effective thermal conductivity of the Fe_3_O_4_-H_2_O magnetic nanofluids for various fractions.

The effective thermal conductivity, *k_nf_*, is shown in Figure 6, both experimentally and theoretically. According to the experimental results for the thermal conductivity of magnetic nanofluids with the volume fraction *f* = 1.0%, the fitting relationship between the depolarization factor, *A_a_* (*A_c_*), of the clusters and magnetic field, *H*, can be obtained. Then, we substitute the field-dependent *A_a_* (*A_c_*) into Equations (2) and (3) to calculate the effective thermal conductivity of magnetic nanofluids with other volume fractions such as *f* = 0.2% and 0.5%. We observe that *k_nf_* exhibits non-monotonic behavior with increasing magnetic field strength. It increases, reaches the maximal value at the saturation magnetic field of about 800 Gs, and then decreases. The thermal conductivity of magnetic nanofluids with *f* = 1.0% is 0.6 W/m·K and 0.95 W/m·K under the absence and presence of an 800 Gs magnetic field. Physically, when there is no applied magnetic field, the magnetic nanoparticles are uniformly distributed within the base fluid, and the magnetic nanoparticles are in Brownian motion, resulting in small thermal conductivity. In the presence of the applied magnetic field, an obvious enhancement of thermal conductivity can be observed because the magnetic nanoparticles form chain-like clusters under the action of the magnetic field, and the heat conduction can be easily transferred through the clusters. Moreover, when the magnetic field strength is further increased and more significant than the saturation one, many parallel chains in the magnetic become tightly packed and form thicker clusters of aggregates, leading to a weak enhancement of thermal conductivity. Moreover, regarding the effect of the volume fraction, *k_nf_* increases monotonically with the increase in *f*. The theoretical results are in good agreement with our experimental data. We further compare our results with those in Ref. [17]. The effective thermal conductivity of Fe_3_O_4_ magnetic nanofluids is found to be increased monotonically with an increase in the magnetic field strength [17]. Here, we predict that when the magnetic field increases, the effective thermal conductivity is increased first, reaches the maximum at the saturation field strength, and then is decreased. Meanwhile, the thermal conductivity enhancement of the 1.0% Fe_3_O_4_ magnetic nanofluids is 58% at the saturation magnetic field of 800 Gs, which is higher than that under the magnetic field in Ref. [17].

### 3.2. Photothermal Properties

The theoretical simulation part of this section is based on the model of Tyagi et al. and Otanicar et al. the direct-absorption solar collectors of nanofluids [38,39]. To calculate the temperature of magnetic nanofluids, one should resort to the energy balance equation, which is expressed as follows:

(5) $$\rho_{nf}C_{p,nf}\frac{\partial T}{\partial t}=k_{nf}\frac{\partial^{2}T}{\partial y^{2}}-\frac{\partial q_{rad}}{\partial y},$$

where *ρ_nf_* and *C_ρ_*_,__*nf*_ are, respectively, the density and specific heat of the magnetic nanofluids, *y* is the depth of the collector, and *T* is the temperature of the nanofluids. Moreover, *q_rad_* is the radiant heat flux given by the following:

(6) $$q_{rad}=\iint_{\lambda ,\phi }I_{\lambda }d\phi d\lambda .$$

**Figure 7:**
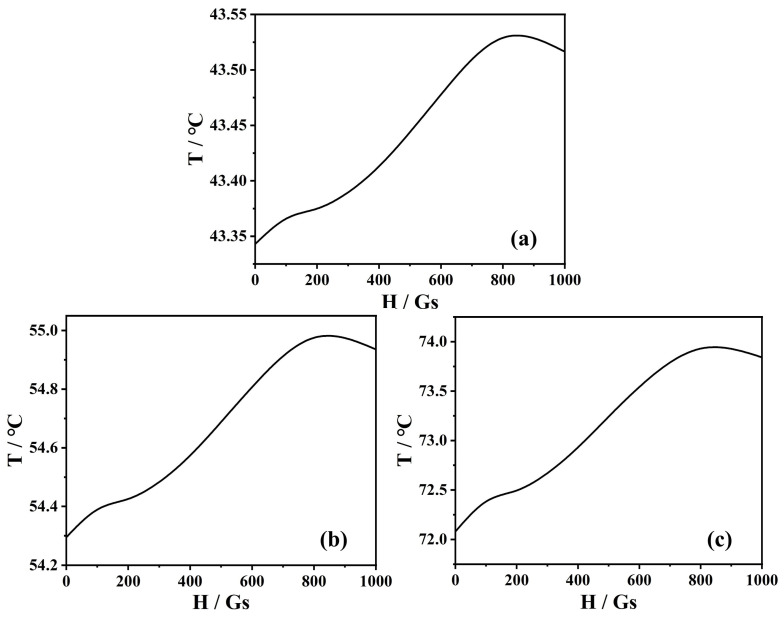
Theoretical results for the temperature of the magnetic nanofluids as a function of the applied magnetic field for (**a**) *f* = 0.2%, (**b**) *f* = 0.5%, and (**c**) *f* = 1.0%.

In Equation (6), *I_λ_* is the intensity distribution within the solar collector, which can be calculated using the radiation transfer equation [38,39]. Figure 7 shows the temperature of the magnetic nanofluids as a function of the magnetic field. As the magnetic field strength increases, the particles form aggregates, and the effective thermal conductivity increases. When the magnetic field strength equals the saturation of 800 Gs, the magnetic nanofluid with *f* = 1.0% can reach a maximal temperature of 73.9 °C. Consequently, heat conduction is accelerated in the magnetic nanofluids, and the temperature of magnetic nanofluids is continuously enhanced. However, when the magnetic field strength is further increased above the saturation magnetic field strength, the chain aggregates become tighter and thicker, decreasing thermal conductivity. In this connection, one observes that the temperature declines too. Hence, we conclude that the magnetic field strength affects the thermal conductivity and thus plays a vital role in the photothermal properties of magnetic nanofluids.

**Figure 8:**
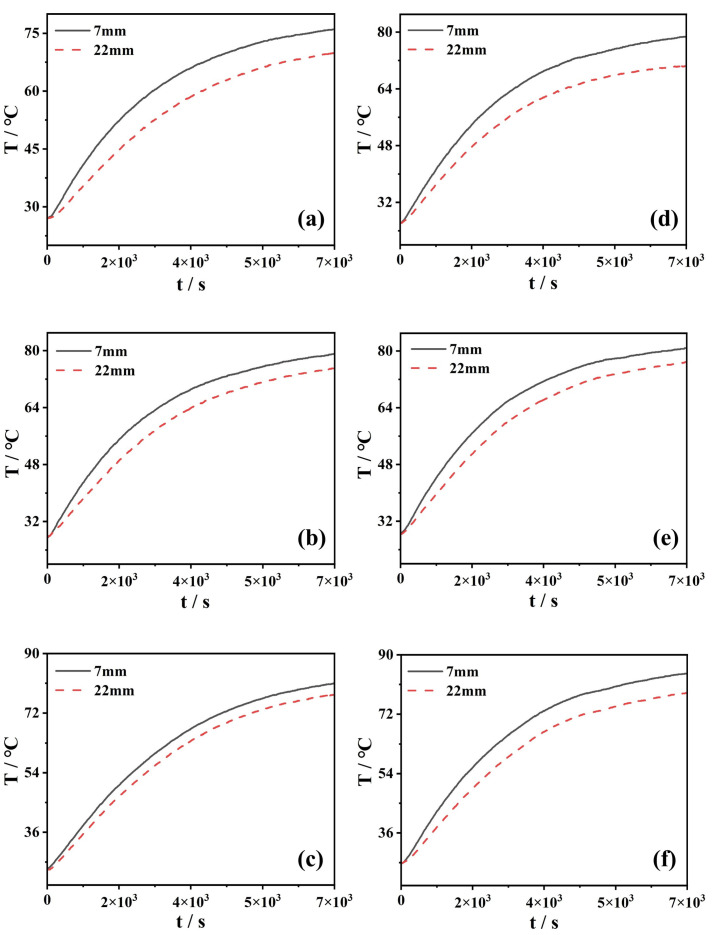
Temperature of Fe_3_O_4_ magnetic nanofluids with different volume fractions: (**a**) 0.2%, (**b**) 0.5%, and (**c**) 1.0% for *H* = 0 Gs, and (**d**) 0.2%, (**e**) 0.5%, and (**f**) 1.0% for *H* = 700 Gs.

Figure 8 shows the experimental results of the temperature of Fe_3_O_4_-H_2_O magnetic nanofluids with various volume fractions. The temperature in the magnetic nanofluid’s upper section (*h* = 7 mm) is generally higher than that in the lower section (*h* = 22 mm). Moreover, the rate of the temperature rise of the upper section is greater than that of the lower section. As for deionized water, the temperature at 7 mm and 22 mm from the light surface can be 70.8 °C and 67.1 °C after 2 h of exposure to light (not shown here). In our case, the temperatures of Fe_3_O_4_-H_2_O magnetic nanofluids in the absence of the magnetic fields are higher than those of deionized water (see Figure 8a–c). In addition, the temperature increases along with the volume fraction of magnetic nanofluids. For instance, the Fe_3_O_4_-H_2_O magnetic nanofluid with 1.0% can reach the maximal temperature of 81 °C, which is increased by 14.41% compared to the one in deionized water. The temperature of the Fe_3_O_4_ nanofluid increases with time, as reported in Ref. [17]. However, the maximum temperature increase in the Fe_3_O_4_ nanofluid is 22.7 °C [17], which is lower than that in our results.

When the external magnetic field *H* = 700 Gs is applied, the temperature of Fe_3_O_4_-H_2_O magnetic nanofluids is further increased for the given volume fractions (see Figure 8d–f). For instance, the magnetic nanofluid with the volume fraction *f* = 1.0% presents us with the highest temperature, up to a maximum of 4.07%, compared to the one without a magnetic field.

**Figure 9:**
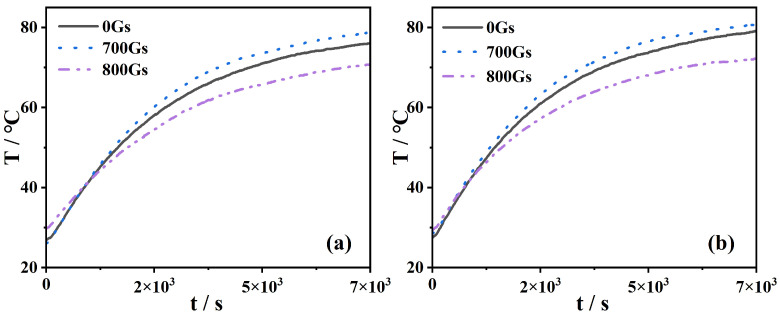
Experimental results on the temperature of magnetic nanofluids at *h* = 7 mm under different magnetic fields for (**a**) *f* = 0.2% and (**b**) *f* = 0.5%.

Experimentally, if the magnetic field strength continues to increase, the temperature of the magnetic nanofluid decreases. We observe that the temperature of magnetic nanofluids under *H* = 700 Gs is higher than that of magnetic nanofluids under *H* = 800 Gs, as shown in Figure 9. Actually, the temperature of magnetic nanofluids under *H* = 800 Gs is even lower than that of a magnetic nanofluid without a magnetic field. The possible explanation is that an excessive magnetic field strength will lead to severe aggregate phenomena, and aggregated clusters cannot absorb light energy very well, which tends to reduce the conversion of light energy into thermal energy. Therefore, we conclude that there is an optimal magnetic field strength to achieve the maximal temperature.

The magnetic nanofluid possesses a higher temperature than deionized water does under solar irradiation and has a much more superior photothermal conversion capability. The equation for photothermal conversion efficiency is written as follows [40]:

(7) $$\eta =\frac{mC_{p}(T_{f}-T_{a})}{AG\Delta t},$$

where *T_a_* (*T_f_*) is the initial temperature (final temperature after two hours) of the magnetic nanofluids, *m* = 75 g is the mass of the magnetic nanofluids, *C_p_* is the specific heat of the nanofluids, *G* = 900 W/m^2^ is the irradiation intensity of the artificial light source, *A* is the irradiation area, and the whole illumination time, Δ*t*, is 2 h.

**Figure 10:**
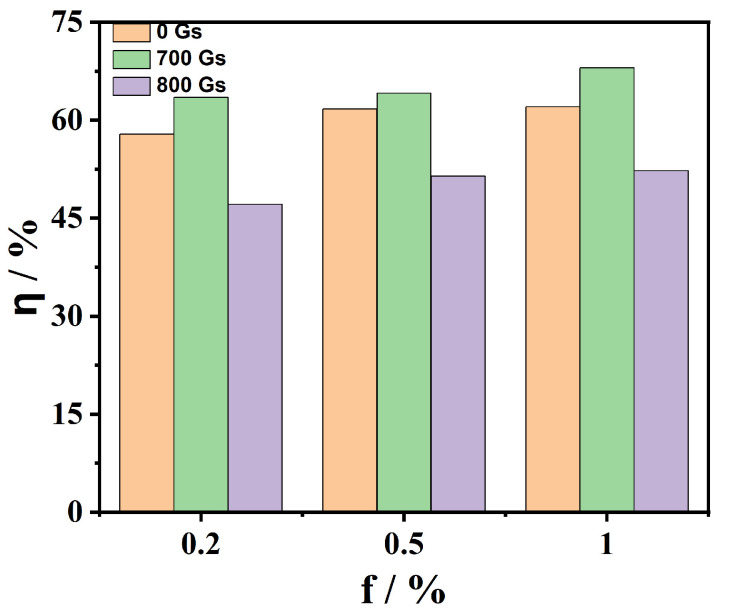
The photothermal conversion efficiency of Fe_3_O_4_-H_2_O magnetic nanofluids with different volume fractions.

According to the temperature of the experimental results and Equation (7), the photothermal conversion efficiency of magnetic nanofluids is calculated, as shown in Figure 10. It is evident that the conversion efficiency of magnetic nanofluids with *f* = 1.0% is higher than that with *f* = 0.5% and *f* = 0.2%. The conversion efficiency of Fe_3_O_4_-H_2_O magnetic nanofluid with *f* = 1.0% increases up to 20.25% compared to that with deionized water. The main reason is that the more significant the volume fraction is, the more nanoparticles are dispersed in the base liquid per unit volume, resulting in more absorption of light energy by the particles. As a consequence, the photothermal conversion efficiency is higher. When the applied magnetic field is considered, the photothermal conversion efficiency of Fe_3_O_4_-H_2_O magnetic nanofluids is further increased, and it reaches a maximum under the saturated magnetic field of 700 Gs. Furthermore, it is found that the maximal conversion efficiency of magnetic nanofluids with *f* = 0.2% under the action of *H* = 700 Gs increases to 9.7% compared to that without a magnetic field. We note that in Ref. [19], the photothermal conversion efficiency of the magnetic nanofluid was improved with the increase in the magnetic field strength, and the optimal magnetic field strength was predicted (but not observed). Notably, we observe such behavior experimentally. Furthermore, the photothermal conversion efficiency of magnetic nanofluids was more than 10% greater than that of the nanofluids in Ref. [17].

### 3.3. Sensitivity and Error Analysis

Due to the various errors in experiments, it is necessary to determine the uncertainty of experimental results via the measurement deviations of multiple parameters. In the experiments, the precision of the temperature meter (DC5508U) is ±0.2%, and the thermal conductivity meter’s (HCDR-S) accuracy is ±3%. According to Equation (7), the relative error of the photothermal conversion efficiency in the indirect measurement can be calculated with the following equation [18,30,41,42]:

(8) $$E_{\eta }=\sqrt{{\left(\frac{\sigma_{m}}{m}\right)}^{2}+(\frac{\sigma_{C_{p}}}{C_{p}}{)}^{2}+\frac{\sigma^{2}{}_{\left(T_{f}-T_{a}\right)}}{{\left(T_{f}-T_{a}\right)}^{2}}+{\left(\frac{\sigma_{A}}{A}\right)}^{2}+{\left(\frac{\sigma_{G}}{G}\right)}^{2}+(\frac{\sigma^{2}{}_{\Delta t}}{\Delta^{2}t})}.$$

By analyzing the absolute errors of various single directly measured parameters such as *σ_m_*/*m* ≤ 0.14%, *σ_Cp_*/*C_p_* ≤ 0.1%, *σ*_Δ*t*_/Δ*t* ≤ 0.2%, *σ_A_*/*A* ≤ 0.12%, *σ_G_*/*G* ≤ 2%, and _*σ*(*Tf*__−*Ta*)_/(*T_f_* − *T_a_*) ≤ 0.2%, we obtain the maximal uncertainty for the photothermal conversion efficiency of 2.03%.

## 4. Conclusions

In conclusion, we have involved the droplet–droplet mixing technique to prepare Fe_3_O_4_-H_2_O magnetic nanofluids. Since our method changes the dispersion pattern from the ordinary liquid–liquid mixing pattern to droplet–droplet one, the prepared magnetic nanofluids maintain good stability within 30 days. Experimental results indicate that both the thermal conductivity and the photothermal properties of magnetic nanofluids exhibit nonmonotonic variation, which includes the rise, the maximum, and the decrease with the increase in the magnetic field strength. On the other hand, the homogenization method is adopted to investigate the effective thermal conductivity and the photothermal conversion efficiency of the magnetic nanofluids, and theoretical predictions agree well with the experimental results. Therefore, the applied magnetic field can enhance the thermal conductivity and the photothermal performance of the Fe_3_O_4_-H_2_O magnetic nanofluids. For the magnetic nanofluid of a 1.0% concentration, the maximum enhancement in thermal conductivity can be 58%, and the conversion efficiency increases up to 20.25% compared to that with deionized water. Physically, the increase in thermal conductivity and photothermal performance is mainly attributed to the effective conduction of heat through the chain-like structures formed under a magnetic field. The enhanced properties and tunability of magnetic nanofluids can lead to some potential applications, such as direct absorption solar collectors, heat exchangers, and automobile radiators.

## Data Availability

The data presented in this study are available upon request from the corresponding author.
